# Help-seeking behaviours, barriers to care and self-efficacy for seeking mental health care: a population-based study in Rwanda

**DOI:** 10.1007/s00127-015-1130-2

**Published:** 2015-10-03

**Authors:** Aline Umubyeyi, Ingrid Mogren, Joseph Ntaganira, Gunilla Krantz

**Affiliations:** Department of Epidemiology and Biostatistics, School of Public Health, College of Medicine and Health Sciences, University of Rwanda, Kigali, Rwanda; Department of Public Health and Community Medicine, Section for Epidemiology and Social Medicine, Sahlgrenska Academy at the University of Gothenburg, Gothenburg, Sweden; Department of Clinical Sciences, Obstetrics and Gynecology, Umeå University, Umeå, Sweden

**Keywords:** Help seeking, Barriers to health care, Self-efficacy for seeking mental health care, Depression, Suicidality

## Abstract

**Purpose:**

Mental disorders commonly affect young people but usually go unrecognized and untreated. This study aimed to investigate help-seeking behaviours, barriers to care and self-efficacy for seeking mental health care among young adults with current depression and/or suicidality in a low-income setting.

**Methods:**

This cross-sectional study used two sub-populations: a sub-sample of those suffering from current depression and/or suicidality (*n* = 247) and another of those not suffering from these conditions and not suffering from any other mental condition investigated (*n* = 502). Help-seeking behaviours, barriers to care and self-efficacy for mental health care seeking were measured among those suffering from current depression and/or suicidality (*n*, %). Logistic regression was used to identify risk factors for experiencing barriers to care. Self-efficacy for seeking mental health care was compared between men and women in the two sub-populations.

**Results:**

Of the 247 men and women with current depression and/or suicidality, 36.0 % sought help at a health care unit and 64.0 % from trusted people in the community. Only six people received help from a mental health professional. The identified barriers were mainly related to accessibility and acceptability of health services. For the population suffering from current depression and/or suicidality, the self-efficacy scale for seeking mental health care suggested a low confidence in accessing mental health care but a high confidence in respondents’ ability to successfully communicate with health care staff and to cope with consequences of seeking care.

**Conclusion:**

The current study clearly highlights young adults’ poor access to mental health care services. To reach universal health coverage, substantial resources need to be allocated to mental health, coupled with initiatives to improve mental health literacy in the general population.

## Background

Young adulthood represents the peak age for the start of mental health problems, yet it is a period of crucial importance for the establishment of emotional well-being in adult life [1]. Mental disorders including depression and suicide attempts are reported to be prevalent among young people worldwide, with the majority being women [2, 3]. Despite the seriousness of mental disorders among young people [4], they remain the least likely to seek help when emotional problems arise, even though these problems can hinder their everyday functioning and well-being [5, 6]. Findings from both low and high income countries show that poor mental health literacy, stigma, embarrassment, ignorance of own illness and financial constraints are key barriers to care for mental problems [3, 6, 7].

In the last decades, the right to the highest attainable standard of mental health has been valued in international human rights treaties at the global level [8]. However, studies have shown that people with mental disorders are often disregarded and discriminated against, which risks the fulfilment of their Right to Health but the standards of mental health care are also not always equitable, i.e., available, accessible, acceptable, and of a good quality for all [3, 8, 9].

Seventeen years have passed since the Rwandan genocide took approximatively one million lives and destroyed the country’s economy and its health infrastructure [10]. Demolitions of health facilities, supply chains for medications and equipment have obstructed the health system for years [10, 11]. After the genocide, Rwanda formalized the irrefutable “Right to Health” in the Rwandan constitution [12]. To ensure that everyone in the society has the right to the highest attainable standard of physical and mental health, Rwanda’s first step towards universal health coverage was to extend the community-based health insurance (mutual health insurance), basically grounded on the concept of solidarity [12]. To improve the quality of care, Rwanda started the extension of the performance-based pay initiative in 2004–2005 after its initial experiences in 2001–2002 in some districts of the country [13]. In the last decade, an improvement of general health in the Rwandan population has been noticeable with improved access to care [12], evidenced for example by a substantial reduction in under-5 mortality and an increase in deliveries assisted by a trained health professional just to cite a few examples [10, 13]. However, such a positive change did not occur for all health conditions, and some health areas still need to be improved [11, 14].

The integration of mental health in the primary health care as recommended by the World Health Organization [15] is still in progress [16]. However, the accessibility to mental health services is limited due to the restricted number of facilities specialized in mental health care and to a shortage of mental health professionals. District hospitals are commonly staffed with a mental health nurse and a clinical psychologist, while health centres lack trained personnel in mental health. In total, the country has only two mental health referral facilities, five psychiatrists [17], and the budget dedicated to mental health in Rwanda has been 1 % of the national health budget in the past years [16].

Previous studies from Rwanda point at long distance to health facilities [18] and inability to pay for health services as common barriers to access care [12, 14].

There has been extensive theoretical and explorative research on help-seeking behaviours and barriers to care among young adults from middle and high income countries, but studies are scarce on people’s help-seeking patterns in relation to mental health problems in low-income settings. Further, there are no studies on the coping ability of poor, depressed young men and women in relation to help seeking behaviours.

The aim of this study was to investigate help-seeking behaviours and barriers to seeking mental health care services among men and women with current depression and/or suicidality. Furthermore, the self-efficacy in overcoming barriers to mental health services were investigated [19]. The theoretical concept of the “Right to Health” was used as a guiding framework in this study [8, 20].

This study forms part of a larger project on violence and other traumatic episodes, mental health and barriers to care among young men and women, *The Rwandan Violence, Mental Health and Barriers to Care project* (*RwVMHBC*-*project*).

## Methods

### Study design, study population and sample size

A cross-sectional study was conducted on a representative sample of young adults aged 20–35 years [2]. The sample size calculation from the entire RwVMHBC project has been previously described [2]. In all, 917 households were included, with 440 men and 477 women and two refusals to participate. To find households for inclusion in the survey, a multi-stage random sampling was used. First, of 3512 existing villages in the Southern Province of Rwanda, 35 villages were randomly selected. Secondly, households were selected proportionate to the total number of households in each village and the study participants to be interviewed were randomly selected among eligible people in each household i.e., men and women aged between 20 and 35 years. Two sub-samples from the entire RwVMHBC project sample (*n* = 917) were used in the current study. The first sub-sample consisted of 247 subjects, 78 men and 169 women, who reported current depression and/or suicidality. This sub-sample was used in all analyses with exception of one analysis exploring help-seeking behaviours where we considered only those who felt the need to seek care (*n* = 150). The second sub-sample consisted of men and women without any of the mental disorders investigated (*n* = 502), this population was used only for comparison purposes in the final analysis to explore the pattern of self-efficacy for seeking mental health care items in the sub-two populations.

### Data collection procedures

The data collection was carried out in the Southern Province in 2011–2012, by 13 experienced clinical psychologists from the School of Public Health, University of Rwanda. A questionnaire was developed, piloted after its translation into Kinyarwanda, and revised accordingly. Face to face interviews were performed.

### Measurements

*Help-seeking behaviour* in the current study was defined as any action of energetically seeking help from the health care services or from trusted people in the community and includes understanding, guidance, treatment and general support when feeling in trouble or encountering stressful circumstances [21].

*The perceived need for mental health care* was investigated by asking the question: “have you ever been so emotionally troubled that you felt a need to seek help?” with a yes or no option. If the participant answered yes, the follow up question was whether they received help from health care services and/or from any other source. The following question asked where they went to seek help within the health care sector: “*to a health centre or district hospital (nurse, medical doctor* etc.)”, “to the district hospital to see mental health professional”, “to a mental health clinic or hospital” and “to a private clinic”. A summary measure for seeking help in any of these health care units was constructed and dichotomized into seeking mental care from a health care unit, as opposed to not doing so. One question asked about other sources of support/help with the options: “wife/partner”, “parent”, “other relative”, “friend”, “teacher”, “religious person”, “community health worker” and “traditional healer”. A summary measure of support from other sources was created and dichotomized as any experience of support or help from the trusted people in the community cited above versus no such experience.

The help-seeking behaviours, the need for mental health care and the barriers to care items were constructed based on previous studies [5, 22–25].

The barriers to mental health care were measured by asking the reasons of not seeking mental health care and these barriers were grouped into structural (five items), individual (ten items) and stigma-related (five items) barriers to care. For example, the structural barriers was explored by asking if someone did not seek care because of the following reasons: “it was too far to get there”, “there was no transport available”, “I could not afford to pay the transport costs”, “I could not pay the fee at the health care centre”, “I have no health insurance”. A summary measure for each type of the barriers (structural, individual and stigma related) was constructed and finally dichotomised into exposure to any of the barrier item, as opposed to no exposure.

To assess the study population’s confidence level to master various barriers to mental health care, their self-efficacy in seeking mental health care was investigated. A recently constructed scale called “Self-efficacy scale for seeking mental health care” with its two sub-scales constructed by Moore et al. [26] was used. The construct of this scale builds on Bandura’s recommendations on how to build self-efficacy scales [27]. Bandura’s theoretical basis was that items should correctly mirror the construct of self-efficacy and that a good self-efficacy scale should accurately reflect the domain of functioning that is being assessed. Therefore, the constructed self-efficacy scale for seeking mental health was adapted based on a review of previous literature on access to care [23, 28, 29] and mental health literacy [30], particularly knowledge, insight and ability to follow through with treatment recommendations as well as psychological factors, including stigma [31]. The constructed scale was linked first to confidence in knowing how to access mental health care and how to communicate with health care staff, forming the self-efficacy knowledge sub-scale (SE-Knowledge). The second sub-scale on how to successfully cope with social and interpersonal consequences of seeking care, formed the self-efficacy coping sub-scale (SE-Coping) [26].

To measure the respondents’ confidence and coping ability when seeking mental health care, each participant was asked to rank the items between one and ten. A summary measure of low (i.e., 1–3), medium (i.e., 4–6) and high (i.e., 7–10) confidence was then constructed; only the low and high categories are presented in table.

To assess mental health status, including current depression (i.e., major depressive episode over the past 2 weeks) and suicidality, the MINI International Neuropsychiatric Interview version 5.0.0, developed from the DSM-IV criteria, was used. Validation studies show that the MINI has similar validity and reliability properties as does the World Health Organization CIDI (Composite International Diagnostic Interview for ICD-10) instrument [32]. The major depressive episode section of the MINI starts with two screening questions corresponding to the main criteria of the disorder and ends with a diagnostic conclusion indicating whether the criterion was met or not. For suicidality, consisting of six questions related to symptoms, diagnosis was reached when at least one was met [2].

The socio-demographic and psycho-social variables were tested as independent risk factors for structural, individual and stigma-related barriers. Age was categorized as a three category variable (20–24, 25–29, and 30–35 years). *Marital status* was divided into married/cohabiting and divorced/widowed and single. The *educational level* was described as a three group variable (secondary school/university, complete primary/vocational training and incomplete primary). As a proxy for socio-economic status of households, the available *assets in the household* (radio, television set, refrigerator, bicycle, motorcycle, car, mobile phone and computer) were merged and dichotomized into having at least one of the items versus having none of the items.

### Statistical analysis

Differences between men and women in terms of socio-demographic factors, help-seeking behaviours and perceived barriers to health care services were assessed by the Pearson’s Chi square test or the Fisher’s exact test for independence for all categorical variables. Logistic regression was used to estimate only predictors of structural and individual barriers but no such analysis was performed for stigma related barriers due to the few cases. To assess the internal consistency of the constructed self-efficacy scale for seeking mental health care, the Cronbach’s α was computed for each subscale, SE-Knowledge and SE-Coping and for the total scale. Cronbach’s *α* coefficients showed a good internal consistency for the self-efficacy total scale for seeking care for a mental disorder (0.901 for men and 0.865 for women), with its two subscales on SE-Knowledge (*α* = 0.877 and 0.836 for men and women respectively) and SE-Coping (*α* = 0.841 and 0.836, for men and women respectively). IBM SPSS Statistics version 20 was used for all statistical analyses.

We used the Mann–Whitney *U* test to obtain *p* values comparing estimates for men and women in relation to self-efficacy Knowledge and Coping items, for the population with current depression and/or suicidality and for the population without any mental disorder respectively.

### Ethical statement

The study was authorized by the National Institute of Statistics of Rwanda (No. 1043/2011/10/NISR) and approved by the Rwanda National Ethics Committee (Review Approval Notice No. 165/RNEC/2011). Complete anonymity and confidentiality were assured, and only one interview per household was done. Participation in the study was voluntary, and a written informed consent was obtained from all participants prior to their inclusion in the study.

## Results

### Socio-demographic and psycho-social factors

The majority of the sub-population suffering from current depression and/or suicidality (*n* = 247) had an incomplete primary education (men: *n* = 57, 76.0 %, women: *n* = 119, 71.3 %). Similarly, the socio-economic status, assessed by available assets in the household, was overall low with almost equal proportions of men and women having at least one of the household assets investigated (men: *n* = 48, 61.5 % and women: *n* = 100, 59.2 % respectively). However, more than one-third of the population had none of the household assets inquired about (men: *n* = 30, 38.5 %, women: *n* = 69, 40.8 %) (Table 1).

**Table 1:** Socio-demographic and psycho-social characteristics of the respondents for the total population (*n* = 917) and the population with depression and/or suicidality (*n* = 247)

	Total population (with and without depression and/or suicidality)					Population with depression and/or suicidality				
	Men (*n* = 440)		Women (*n* = 477)		*p* value*	Men (*n* = 78)		Women (*n* = 169)		*p* value*
	*n*	%	*n*	%		*n*	%	*n*	%	
Respondents’ characteristics										
1. Age groups										
20–24 years	148	33.8	127	27.0	0.050	23	29.5	51	30.5	0.666
25–29 years	144	32.9	156	33.2		31	39.7	57	34.1	
30–35 years	146	33.3	187	39.8		24	30.8	59	35.3	
2. Marital status										
Married or cohabiting	236	53.8	342	72.3	0.000	41	53.2	116	68.6	0.000
Divorced or widowed	2	0.5	33	7.0		1	1.3	16	9.5	
Single	201	45.8	98	20.7		35	45.5	37	21.9	
3. Education level										
Secondary school or university	50	13.3	67	17.0	0.006	8	10.7	27	16.2	0.531
Complete primary school or vocational training	105	28.0	73	18.6		10	13.3	21	12.6	
Incomplete primary school	220	58.7	253	64.4		57	76.0	119	71.3	
Partner’s characteristics										
4. Ever been to school										
Attended school	211	83.7	287	75.9	0.021	38	79.2	95	72.0	0.443
Partner no schooling	41	16.3	91	24.1		10	20.8	37	28.0	
Household (HH) characteristics										
5. Assets in the household										
Have at least 1 of the assets	323	73.4	331	69.4	0.189	48	61.5	100	59.2	0.781
Have none of the HH assets	117	26.6	146	30.6		30	38.5	69	40.8	

* *p* value signifies statistical difference between men and women

### Help-seeking behaviours for mental health care

Of the total of 247 persons who fulfilled the diagnosis of current depression and/or suicidality, 150 people, 43 (55.1 %) men and 107 (63.3 %) women recognized that they had been so emotionally troubled that they felt the need to seek help. Of those 150 people, 54 (36.0 %) sought help from a health care unit while 96 (64.0 %) sought help from people they trusted in the community (Table 2). The first choice for those that sought help from a health care unit was a health centre or a district hospital where they met a health professional, who was not however trained in mental health. Only one man and four women managed to seek help from either a trained mental health professional at the district hospital or at a mental health hospital/clinic.

**Table 2:** Help-seeking behaviours of men and women with depression or suicidality, *n* = 247, 78 men and 169 women

	Total (*n* = 247)		Men (*n* = 78)		Women (*n* = 169)		*p* value*
	*n*	%	*n*	%	*n*	%	
1. Help-seeking for emotional problems?							
Yes	150	60.7	43	55.1	107	63.3	0.262
No	97	39.3	35	44.9	62	36.7	

	Total (*n* = 150)		Men (*n* = 43)		Women (*n* = 107)		*p* value*
2. Seeking help in a health care unit?							
To a health centre or district hospital	38	25.3	8	18.6	30	28.0	
To a district hospital to see mental health professional	5	3.3	1	2.3	4	3.7	
To a mental health clinic/mental hospital	1	0.6	0	0.0	1	0.9	
To a private clinic	11	7.3	5	11.6	6	5.6	
Visited at least one of the above health care units)	54	36.0	13	30.2	41	38.3	0.236
3. Seeking help from others (other source of support)?							
Wife/Partner	11	7.3	2	4.7	9	8.4	
Parent	12	8.0	3	7.0	9	8.4	
Other relative	13	8.7	5	11.6	8	7.5	
Friend	36	24.0	10	23.3	26	24.3	
Teacher	2	1.3	1	2.3	1	0.9	
Religious person	9	6.0	4	9.3	5	4.7	
Community health worker	14	9.3	1	2.3	13	12.1	
Traditional healer or traditional birth attendant	10	6.7	3	7.0	7	6.5	
Visited at least one of the above sources of support	96	64.0	25	58.1	71	66.4	0.477

* *p* value signifies statistical difference between men and women

When seeking help outside the health care units, the first choice was a friend (*n* = 36, 24.0 %) for both men and women, followed by the community health worker in the case of women, while a relative was the second choice for men. The remaining sought help from a parent, a partner, a religious person, a teacher or a traditional healer (Table 2). However, of the 247 men and women (78 men and 169 women), who reported suffering from current depression and/or suicidality, 35 men (44.9 %) and 62 women (36.7 %) did not seek help from any of the above, while 10 men (12.8 %) and 32 women (18.9 %) sought help from both trusted people and from a health care unit.

### Barriers to care

Among the structural level barriers, men and women were most concerned about not being enrolled in a health insurance scheme (12.8 % of men and 19.5 % of women) and not being able to pay the fee (12.8 % of men and 18.9 % of women). The most commonly identified barriers to effective health care services were the individual barriers, for both men and women and these were mainly related to knowledge and attitudes to health care seeking. Of the individual barriers, the most commonly experienced by women was to believe that they ‘would not get proper treatment’, while men reported a belief that ‘the problem would disappear by itself’.

Stigma-related barriers were the least reported, however more commonly reported among men than women even though the difference was not statistically significant. Among the stigma-related barriers, men and women were mainly worried that the health care staff would have negative attitudes towards them or of themselves bringing a bad name to their family due to suffering from a mental disorder (Table 3).

**Table 3:** Barriers to care as perceived by men and women with depression and suicidality, *N* = 247, 78 men

Variables	Total		Men		Women		*p* value*
	*n*	%	*n*	%	*n*	%	
1. Structural barriers							
It was too far away to get there	14	5.7	1	1.3	13	7.7	0.071
There was no transport available	35	14.2	6	7.7	29	17.2	0.051
I could not afford to pay the transport costs	39	15.8	9	11.5	30	17.8	0.262
I could not pay the fee at the health care centre	42	17.0	10	12.8	32	18.9	0.277
I have no insurance	43	17.4	10	12.8	33	19.5	0.212
Summary measure (at least exposed to one of the barriers)	52	21.1	10	12.8	42	24.9	0.043
2. Individual barriers related to knowledge and attitudes							
I did not know where to go for treatment	29	11.7	4	5.1	25	14.8	0.033
I was too embarrassed to discuss my problems with anyone	28	11.3	7	9.0	21	12.4	0.520
I did not believe that I would get proper treatment	37	15.0	6	7.7	31	18.3	0.034
I did not believe that treatment could help me	34	13.8	5	6.4	29	17.2	0.028
I thought my problem was one I should be able to cope with myself	34	13.8	12	15.4	22	13.0	0.692
I thought that the problem would disappear by itself	28	11.3	14	17.9	14	8.3	0.032
I was afraid of the consequences of seeking care (treatment, tests, hospitalization, operations…)	15	6.1	4	5.1	11	6.5	0.781
I did not want any help	12	4.9	2	2.6	10	5.9	0.349
I got help from another source	26	10.5	7	9.0	19	11.2	0.662
Other responsibility such as taking care of children/family members	17	6.9	3	3.8	14	8.4	0.282
Summary measure (at least exposed to one of the barriers)	67	27.1	17	21.8	50	29.6	0.221
3. Stigma-related barriers							
I was afraid that somebody I knew would see me at the health care clinic	6	2.4	4	5.1	2	1.2	0.081
I was ashamed to show others how emotionally troubled I was	9	3.6	4	5.1	5	3.0	0.469
I was afraid that the health care staff would have negative attitudes towards me	11	4.5	5	6.4	6	3.6	0.331
I was afraid to bring bad name to my family if I disclosed to health staff that I felt emotionally troubled	11	4.5	5	6.4	6	3.6	0.331
I did not trust that health staff will keep my problem confidential	7	2.8	4	5.1	3	1.8	0.212
Summary measure (at least exposed to one of the barriers)	14	5.7	6	7.7	8	4.7	0.381

* *p* value signifies statistical difference between men and women

To check for barriers to care predictors among those suffering from current depression or suicidality, associations between different socio-demographic factors and structural and individual level barriers to care were investigated. In the crude logistic regression analyses for women, we found that having a partner with no schooling (crude OR 3.40; 95 % CI 1.50–7.71) and having no assets in the household (crude OR 2.13; 95 % CI 1.05–4.32) displayed statistically significant odds ratios for structural barriers (Table 4). Women’s low education, with a crude odds ratio of 2.10 (0.89–4.93), also suggested an effect but failed to reach statistical significance. No socio-demographic variables were associated with any of the individual barriers investigated for women, and no statistically significant associations for any of the barriers investigated were found for men (Table 4).

**Table 4:** Association between socio-demographic, psychosocial factors and barriers to care for men and women with current depression or suicidality; Crude odds ratios with 95 % confidence intervals, *N* = 247, Men, *n* = 78 and Women, *n* = 169

Variables	Men				Women			
	Structural barriers		Individual barriers		Structural barriers		Individual barriers	
	*n* (%) with exp.	OR (95 % CI)	*n* (%) with exp.	OR (95 % CI)	*n* (%) with exp.	OR (95 % CI)	*n* (%) with exp.	OR (95 % CI)
Respondent’s age								
20–24 years	4 (17.4)	1	5 (21.7)	1	10 (19.6)	1	15 (29.4)	1
>24 years	6 (10.9)	0.58 (0.15–2.29)	12 (21.8)	1.01 (0.31–3.27)	32 (27.6)	1.56 (0.70–3.48)	35 (30.2)	1.04 (0.50–2.13)
Respondent’s education								
Complete primary or secondary school or vocational training or university	9 (15.8)	1	13 (22.8)	1	34 (28.6)	1	36 (30.3)	1
Incomplete primary or no schooling	1 (5.6)	3.19 (0.38–27.06)	4 (22.2)	1.03 (0.29–3.69)	8 (16.0)	2.10 (0.89–4.93)	14 (28.0)	1.12 (0.54–2.32)
Partner’s ever been to school								
Yes	5 (13.2)	1	6 (15.8)	1	19 (20.0)	1	28 (29.5)	1
No	2 (20.0)	1.65 (0.27–10.1)	3 (30.0)	2.29 (0.46–11.43)	17 (45.9)	3.40 (1.50–7.71)	15 (40.5)	1.63 (0.74–3.60)
Assets in the household								
Yes	5 (10.4)	1	9 (18.8)	1	19 (19.0)	1	27 (27.0)	1
No	5 (16.7)	1.72 (0.45–6.53)	8 (26.7)	1.58 (0.53–4.67)	23 (33.3)	2.13 (1.05–4.32)	23 (33.3)	1.35 (0.69–2.64)

### Self-efficacy for seeking mental health care

Finally, we decided to investigate the self-efficacy related to seeking health care for men and women with a mental disorder (current depression or suicidality) and for men and women not reporting any of the investigated mental condition (*n* = 502), and a clear pattern evolved.

Among those suffering from current depression and/or suicidality, for the first four items (a–d items) of the Knowledge scale, we found that both men and women had a low confidence in finding a place to get mental treatment, get transportation, pay for transportation and pay for the services. However, women to a higher extent than men exhibited low confidence for all items but not for the item “to get transportation (*p* value 0.053)”. The pattern for those not suffering for a mental disorder was almost similar for the first four items of the Knowledge scale but with no statistical significant difference between men and women.

For the last three items on personal behaviour (e–g items), both men and women suffering from depression and/or suicidality displayed a high confidence, however more women than men in their abilities to tell the staff what is troubling them, understanding the information given to them by the staff and to follow treatment recommendations. For the population not suffering from any mental disorder, the patterns were not evident and no difference was found between men and women for the three items.

When comparing men’s and women in the population suffering from current depression and/or suicidality (h–k items) the population, more women than men displayed high confidence. For those not suffering for mental disorders, the coping ability was similar for men and women (Table 5).

**Table 5:** Self-efficacy for seeking care, among men and women suffering from depression and/or suicidality as compared to those not suffering from any mental condition

Self-efficacy for seeking care	Population not reporting any of the mental disorders investigated* (*n* = 502, men: *n* = 271 and women, *n* = 231)					Population with depression and/or suicidality (*n* = 247, men: *n* = 78 and women, *n* = 169)				
	Men		Women		*p* value**	Men		Women		*p* value**
If you need mental health care, I feel confident in my ability to:	Low confidence	High confidence	Low confidence	High confidence		Low confidence	High confidence	Low confidence	High confidence	
	*n* (%)	*n* (%)	*n* (%)	*n* (%)		*n* (%)	*n* (%)	*n* (%)	*n* (%)	
1. Knowledge										
Find a place to get mental health treatment	95 (35.4)	71 (26.5)	100 (43.7)	75 (32.8)	0.185	29 (37.7)	23 (29.9)	96 (57.5)	42 (25.1)	0.007
Get transportation to mental health care service	111 (41.7)	59 (22.2)	112 (48.5)	56 (24.2)	0.208	44 (57.9)	15 (19.7)	104 (62.3)	31 (18.6)	0.053
Pay for the transportation to mental health care service	101 (47.9)	34 (16.1)	99 (49.3)	41 (20.4)	0.183	36 (50.0)	13 (18.1)	100 (69.4)	18 (12.5)	0.006
Pay the fee for the mental health care service, if there is one	135 (51.1)	50 (18.9)	109 (48.7)	34 (15.2)	0.387	44 (59.5)	10 (13.5)	115 (69.3)	22 (13.3)	0.005
Clearly tell the staff what is troubling me	42 (15.8)	74 (27.8)	57 (24.8)	73 (31.7)	0.550	13 (17.1)	28 (36.8)	43 (25.7)	84 (50.3)	0.092
Understand the information given to me by the staff	35 (13.2)	117 (44.0)	31 (13.4)	87 (37.7)	0.983	7 (9.1)	40 (51.9)	18 (10.8)	97 (58.4)	0.018
Be able to follow the treatment recommendations made by the staff	25 (9.3)	142 (53.0)	25 (10.9)	107 (46.7)	0.952	5 (6.6)	43 (56.6)	12 (7.2)	105 (63.3)	0.005
2. Coping										
Cope well with the consequences of seeking care	25 (9.3)	133 (49.4)	25 (10.9)	104 (45.2)	0.554	5 (6.5)	37 (48.1)	13 (7.8)	95 (56.9)	0.019
Cope well with family or friends’ reactions to me seeking mental health treatment	32 (11.9)	137 (51.1)	19 (8.2)	115 (49.8)	0.607	11 (14.3)	38 (49.4)	16 (9.6)	100 (59.9)	0.007
Cope well with the attitudes that the staff may have towards me	28 (10.4)	137 (51.1)	23 (10.0)	114 (49.4)	0.513	7 (9.3)	34 (45.3)	16 (9.6)	110 (65.9)	0.000
Overcome any embarrassment I may have about seeking mental health treatment	32 (12.0)	133 (49.8)	44 (19.0)	111 (48.1)	0.278	12 (15.6)	40 (51.9)	23 (13.8)	101 (60.5)	0.046

* Current or past depression, suicidality, PTSD or generalized anxiety disorders. From the total sample (*n* = 917), those presenting suicidality, PTSD or generalized anxiety disorders (*n* = 166) are excluded from the analysis in this table

** *p* values are calculated on ordinal knowledge and coping variables (a–k items) using the Mann–Whitney *U* test and presents differences between men and women in the respective populations

## Discussion

### Help-seeking behaviours for mental problems

We found that women, but also men, with emotional problems were more likely to seek help from trusted people in the community (64.0 %) than from the formal health care services (36.0 %) as seen also in a study from South Africa [3], even though effective treatments are available. A friend was the most common person to turn to for both men and women, and the community health worker (CHWs) was the next to be confided in by women, while men preferred to turn to a relative. Of those seeking care at a health unit, the majority turned to a health centre or a district hospital while few accessed a mental health professional. This reflects most probably the scarce mental health care resources at hand [33], but also the poor mental health literacy related to when and where to go for professional care for mental problems [6, 7]. Additionally, we also found that more than one third of those suffering from depression/suicidality (*n* = 247) did not report any need for help and did not use any health care unit or informal help seeking. This is consistent with studies suggesting that most of young adults often do not seek help for a mental disorder [34, 35].

In this study, no statistically significant gender differences were found in relation to help-seeking behaviours probably due to a relatively small sample of men suffering from depression and/or suicidality. However, poor help-seeking behaviours were more pronounced in men than in women. This might be explained by the masculinity traits in which “real men” are considered to be physically fit, careless of their health and self-sufficient [36] while young women are more likely to make use of the most trusted people in their social environment to find help, support and advice for mental health problems [37]. A study from Australia, finds that seeking support from other trusted people can provide emotional, informational and instrumental support [38]. However, the same study emphasized that a disadvantage is the risk of being stigmatized and given uninformed information, which may hinder the use of health care for mental problems when needed.

### Barriers to care

In our exploration of the participants’ perceptions related to health care seeking and barriers to care, interesting gender differences were found. Women, to a considerably higher extent than men faced structural barriers, such as problems in accessing health services, especially transport to a health facility, also reported in another study [37]. The plausible explanation is that women have fewer resources, and are not able to make own decisions on how to organize such a transport.

Among the individual barriers to care investigated, mirroring knowledge and attitudes to care seeking, there are some interesting findings suggesting health illiteracy to be more common among women (“did not know where to go for help”) while there was also evidence of distrust in the primary health care services (“did not believe in receiving proper treatment”, “did not believe treatment would help”), which at the same time points at a poor quality of mental health services in the primary health care. Men trusted that the problem would disappear by itself, and trusted own coping ability, a possible indication of avoidance behaviour leading to poorer health care seeking [3, 28, 39] related as well to the stigma associated with such conditions. Thus, these findings suggest low mental health literacy in the population, but also poor accessibility and acceptability that make men and women avoid seeking care at a health facility and rather turn to trusted persons in the community.

Even though the stigma surrounding mental disorders is widespread and a well acknowledged obstacle to help seeking for mental problems [40], we found comparatively low reporting of such barriers.

Additionally, poverty and low educational level were key predictors of structural barriers for women, indicating women’s limited resources in terms of decision-making capacity, finance and knowledge related to health.

### Self-efficacy for seeking mental health care

When exploring self-efficacy for seeking mental health care, we found that men and women suffering from current depression and/or suicidality displayed low confidence about overcoming structural barriers as evidenced when exploring barriers to care. However, they exhibited high confidence about overcoming individual and stigma-related barriers to care. This illustrates the low potential in both men and women to defeat access barriers within the health system, especially when belonging to a poor part of the population. Based on a previous study, which finds that people with low self-efficacy were more likely to experience barriers to care [41], we would have expected that most of women but also men who suffered from a mental condition, would report low confidence related to coping ability as they obviously experienced a variety of social and interpersonal barriers to professional care. We however found the opposite i.e., high confidence in their own ability to cope with social and interpersonal consequences of seeking mental health care. From this, it is understood that women, but also men, felt powerless in terms of the ability to influence structures in the society but believe in their personal strengths i.e., displaying a high self-efficacy in coping.

### Strengths and limitations

The data collection was of a good quality, face to face interviews were performed, in a population-based random sample. Interviews, following a questionnaire, were performed by skilled clinical psychologists and the response rate was high.

To measure the study population’s confidence in overcoming a variety of difficulties in seeking mental health care services, we used a recently constructed scale, on the self-efficacy for seeking mental health care [26]. The internal reliability scale was good and these items were used in a parallel study in South Africa [26]. The South African study used exploratory factor analysis, which clearly indicated the two subscales SE-Knowledge and SE-Coping [26]. For depression and suicidality, the MINI instrument was used, a well-known and validated tool for diagnosing mental disorders.

As this was a cross-sectional study, no conclusion could be drawn on the direction of the associations between socio-demographic factors and structural or individual barriers to care for the population suffering from depression or suicidality. However, other studies have evidenced the causal relationship between poor and less educated people and being less able to seek care when in need [42, 43].

## Conclusion

This study found that men and women suffering from depression and/or suicidality preferred to seek help from a relative or a friend and extremely few ever reached out to mental health services. According to the Rwandan constitution, every citizen has the right to the highest attainable standard of health. However, the current study clearly shows that young people meet a set of access barriers to mental health services. To reach universal health coverage, the mental health services need to be made available, accessible, acceptable and of a good quality. The current study findings stress the need to increase the number of health professionals and secure that the mental health care is of a good quality to meet the population’s right to health. It is further important to improve people’s mental health literacy to be able to recognize mental disorders, take appropriate actions when needed and provide well-built support and advice to friends and family members in need.
